# Effect of Biannual Azithromycin Distribution to Infants on Community Gut Resistome and Microbiome: A Cluster-Randomized, Controlled Trial

**DOI:** 10.4269/ajtmh.25-0238

**Published:** 2025-10-23

**Authors:** Boubacar Coulibaly, Daisy Yan, Ali Sié, Kiemdé Dramane, Mamadou Bountogo, Dah Clarisse, Ouermi Lucienne, Fanny Yago-Wienne, Djeinam Touré, Georges Dimithé, Regina Khassanov, Souleymane Sidibé, Noel Pouorinibé Somé, Lina Zhong, Cindi Chen, Danny Yu, YuHeng Liu, Thomas Abraham, Brittany Peterson, Armin Hinterwirth, Benjamin F. Arnold, Kieran O’Brien, Catherine E. Oldenburg, Elodie Lebas, Thomas M. Lietman, Thuy Doan

**Affiliations:** ^1^ Centre de Recherche en Santé de Nouna, Nouna, Burkina Faso;; ^2^ Francis I. Proctor Foundation, University of California, San Francisco, California;; ^3^ Helen Keller International, Dakar, Senegal;; ^4^ Helen Keller International, Ouagadougou, Burkina Faso;; ^5^ Department of Epidemiology and Biostatistics, University of California, San Francisco, California;; ^6^ Department of Ophthalmology, University of California, San Francisco, California;; ^7^ Institute for Global Health Sciences, University of California, San Francisco, California

## Abstract

Biannual azithromycin administration to preschool children in sub-Saharan Africa improved childhood mortality but selected for antibiotic resistance (AMR). WHO guidelines recommended focusing treatment on infants ages 1–11 months old to reduce mortality while minimizing selection of AMR. The Infant Mortality Reduction by the Mass Administration of Azithromycin study was a double-masked, placebo-controlled, cluster-randomized trial that investigated these WHO guidelines. Health centers from three regions of Burkina Faso were randomized in a 2:1 ratio to receive either biannual azithromycin (67%) or placebo (33%) distribution to children 1–11 months old. A total of 3,524 rectal samples from children ages 1–59 months old from 60 randomly selected communities were included in the analysis. The prespecified primary outcome was the community-level fold change in macrolide resistance determinants between arms at the 24-month time point. Macrolide resistance determinants in the gut of children in communities whose infants received azithromycin did not increase compared with those in communities treated with placebo (1.05-fold change). Similarly, the fold changes for resistance determinants for beta-lactams, metronidazole, sulfonamides, tetracycline, and trimethoprim were 0.99-fold, 1.00-fold, 1.22-fold, 0.96-fold, and 0.96-fold, respectively. At 6 months after the fourth treatment, there were no detectable differences in the microbiome structure (Euclidean permutational multivariate analysis of variance) and Shannon diversity index between treatment arms. These results suggest that biannual azithromycin administration to children 1–11 months old did not lead to a significant long-lasting increase in gut AMR or alterations of the gut microbiomes of children 1–59 months old in the community.

## INTRODUCTION

Mass azithromycin distribution to children ages 1–59 months old has been shown to reduce childhood mortality in multiple randomized, controlled trials.^1^^–^^4^ However, community antibiotic resistance (AMR) to macrolide and nonmacrolide antibiotics has significantly increased.^5^^,^^6^ Stakeholders are struggling with the balance between the benefits of mass drug distribution and the potential harm of increased AMR.^7^

In Macrolides Oraux pour Réduire les Décès avec un Oeil sur la Résistance (MORDOR) I, the age group that may have benefited the most was children ages 1–11 months old, with nearly a 25% reduction in childhood mortality compared with the 14% reduction in the age group of children 1–59 months old.^1^ In 2020, the WHO issued a conditional recommendation to treat infants 1–11 months of age with twice yearly mass distribution of azithromycin in areas of sub-Saharan Africa where there were more than 60 infant deaths per 1,000 live births or more than 80 deaths of children younger than 5 years old per 1,000 live births.^8^ Targeting treatment to the most vulnerable population with the highest therapeutic effect may reduce unnecessary AMR.

The objectives of this double-masked, placebo-controlled, cluster-randomized trial were twofold. One objective was to demonstrate whether biannual azithromycin intervention to infants ages 1–11 months old could be scaled by integrating with the existing “Child Health Days” (CHD) platform in Burkina Faso to achieve a similar mortality improvement benefit. The second objective was to quantify changes in AMR in preschool-aged children. This article describes the gut microbiome and antibiotic end points in children in a selected subset of communities enrolled in the mortality trial.

## MATERIALS AND METHODS

### Trial design.

This was a cluster-randomized, placebo-controlled trial to evaluate the impact of biannual azithromycin integrated into the high-dose vitamin A platform on antimicrobial resistance in preschool-aged children ages 1–11 months old. This trial was part of a larger mortality study that included communities in the Sud-Ouest, Centre-Ouest, and Hauts-Bassins regions of Burkina Faso. In this study, the prespecified 60 communities were randomly selected for rectal sample collection for macrolide and nonmacrolide resistance and microbiome analysis. The trial began in September 2021, and sample collection for this study concluded in January 2024.

### Study setting and recruitment.

This trial was conducted in three regions in Burkina Faso. Before every distribution, a census was performed in all selected communities to identify all children ages 1–11 months old and pregnant women. Before the start of all activities, verbal consent was obtained from the village leaders, and written consent was obtained from children’s parents/guardians. All children ages 1–11 months old were recruited to receive the intervention. Every 6 months for 2 years, community health care workers visited the study area and administered azithromycin/placebo to eligible children ages 1–11 months old. They also administered vitamin A and performed other activities included in the CHD package to all eligible children ages 6–59 months old. Rectal samples were collected from a randomly selected subset of children ages 1–59 months old for resistance monitoring in the same communities at baseline and at 24 months (6 months after the fourth azithromycin distribution) unless communities were lost to follow-up and needed replacement. Individual children were not followed, only specific communities.

### Eligibility criteria.

Children ages 1–59 months old living in one of the participating randomized communities were eligible.

### Intervention.

Treatment of children ages 1–11 months old involved a single 20-mg/kg dose (age-based dosing) of oral azithromycin or placebo as a powder for oral suspension.

### Randomization and masking.

The three intervention regions of Burkina Faso were randomized to either CHD with azithromycin (67%) or CHD with placebo (33%), an allocation of 2:1 that favored azithromycin treatment. Randomization was at the Centre de Santé et de Promotion Sociale (CSPS) or primary health care center catchment area level. The allocation sequence was prepared by the University of California, San Francisco (UCSF) and linked to masked treatment bottles by Pfizer. The placebo was indistinguishable from azithromycin. Participants, study personnel, investigators, and outcome assessors were masked to allocation. Sixty communities were selected for this AMR study (one community per CSPS) with the probability of 2:1 (azithromycin:placebo) and were not stratified in any way by region. An additional 14 communities were selected to serve as potential replacement communities if there was a loss to follow-up among the core 60 communities. A single unmasked statistician and analyst on the UCSF team completed the primary analysis separate from the rest of the investigator team. The analysis team initially used a rerandomized allocation sequence (to break any relationship between treatment and outcomes) until the primary analysis was completed (e.g., table and figure shells were completed and populated with results). After the primary analysis workflow was completed, the team then swapped out the rerandomized sequence with the actual sequence to unmask the trial.

### Sample size calculations.

The sample size was determined using the parameters estimated in the MORDOR trial and standard sample size equations for binary and continuous pooled results.^5^^,^^7^ For the determinants of macrolide resistance in the rectal swab samples, we used the load of AMR genetic determinants estimated by read number and normalized. Based on an estimated intraclass correlation of 0.02 from the 24-month MORDOR I Niger data, 30 specimens from 30 communities per group would provide about 80% power to detect a 1.5-fold increase in the load of macrolide resistance determinants assuming SDs of 3.1 in both groups (on the logarithmic scale) and two-sided alphas of 0.05.

### Sample collection and management.

Forty children (ages 1–59 months old) in each community were randomly selected for sample collection at baseline and at 24 months. Rectal swabs were collected and placed in DNA-RNA Shield Collection Kits (Zymo Research, Irvine, CA) to preserve nucleic acids. Samples were stored on ice in the field and at −20°C in Burkina Faso until shipment to UCSF for longer-term storage at −80°C until sample processing.

### Sample processing and bioinformatics.

All samples were deidentified before processing, and the laboratory personnel were masked to the community, time point, and treatment arm assigned to each sample. The order of processing was randomized by an unmasked analyst, and samples were organized by community and time point and randomly allocated a processing order; 3,524 collected rectal samples were pooled at the community level, with each pool representing an analog average load of that community. Total DNA was extracted from 119 pooled rectal samples (60 pooled communities for baseline and 59 pooled communities for 24 months) using the Zymo Quick DNA-RNA MiniPrep Extraction Kits (Zymo Research) per the manufacturer’s instructions and prepared for DNA sequencing libraries using the New England Biolabs NEBNext Ultra II DNA Library Prep Kit (NEB, Ipswich, MA). Then, it was amplified with 10 polymerase chain reaction cycles and sequenced on the NovaSeq X instrument (Illumina, San Diego, CA) using 150-nucleotide paired-end sequencing.^6^^,^^9^

Sequencing reads were analyzed for microbiome and AMR determinants as previously described.^5^^–^^7^ Briefly, host reads were removed, and nonhost reads were quality filtered and passed onto Kraken2 to align to the entire National Center for Biotechnology Information nonredundant collection that was downloaded on November 8, 2022 for subsequent microbiome analysis.^10^ For AMR determinants analysis, the nonhost reads were aligned to the MEGARes reference antimicrobial database (v. 3.0.0) using Burrows-Wheeler Aligner with default settings.^11^^,^^12^ Each identified AMR determinant was grouped at the class level (such as macrolide, aminoglycosides, or beta-lactams) using Resistome Analyzer (https://github.com/cdeanj/resistomeanalyzer) and normalized by the total number of nonhost reads in the respective pooled sample for further statistical analysis. Macrolide resistance determinants belong to the macrolide–lincosamide–streptogramin (MLS) class.

## STATISTICAL ANALYSES

All comparisons were made at the community (village) level. Antimicrobial resistance determinants were specified at the class level. For comparisons of MLS resistance determinants, *P*-values were not corrected for multiple comparisons. For comparisons of non-MLS antimicrobial determinants, *P*-values were corrected for multiple comparisons using the Benjamini–Hochberg false discovery rate procedure.

The prespecified primary outcome for rectal swabs was differential changes in antimicrobial classes between treatment arms at 24 months. Fifty-nine communities were included in this analysis given that this was the number of communities sampled at 24 months. Here, unadjusted *P*-values were obtained through the Wald test. For MLS resistance only, the *P*-value was permuted with 10,000 replicates by shuffling treatment assignments of each community without replacement.

As a sensitivity analysis, analysis of covariance was performed on normalized reads of macrolide resistance determinants to obtain the mean difference in normalized reads between treatment arms at 24 months while adjusting for normalized reads at baseline. The same was performed for nonmacrolide resistance determinants as a secondary analysis. Here, *P*-values were permuted with 10,000 replicates as described above for all antimicrobial classes. False discovery rate corrections for comparisons, excluding macrolide resistance, were applied to permuted *P*-values. Forty-nine communities were included in this analysis given that this was the number of communities that were sampled at both baseline and 24 months.

Microbiome analyses were performed to assess differences in the gut microbiome between treatment arms and time points at the species level. To investigate differences in bacterial community structure, permutational multivariate analysis of variance (PERMANOVA) was performed on both Manhattan distance (L^1^ norm) and Euclidean distance (L^2^ norm) between treatment arms with 10,000 replicates. To investigate differences in alpha diversity or species richness, the Shannon diversity index and the inverse Simpson diversity index were calculated and exponentiated to express the effective number of species. The Simpson diversity index alone is a measure of evenness, and its inverse provides a species richness measure. The Wilcoxon rank sum test was used to test differences in diversity between treatment arms at baseline and 24 months.

All analyses were conducted in R v. 4.3.1 (R Foundation, Vienna, Austria). Differential changes in antimicrobial resistance determinants were obtained using DESeq2.^13^ Analysis of covariance models were fit using the “lm()” function in the “stats” package.^14^ Permutational multivariate analysis of variance models were fit using the “adnois2()” function in the “vegan” package.^15^ Diversity measures were calculated using the “vegan” package.

## RESULTS

**Figure 1.**
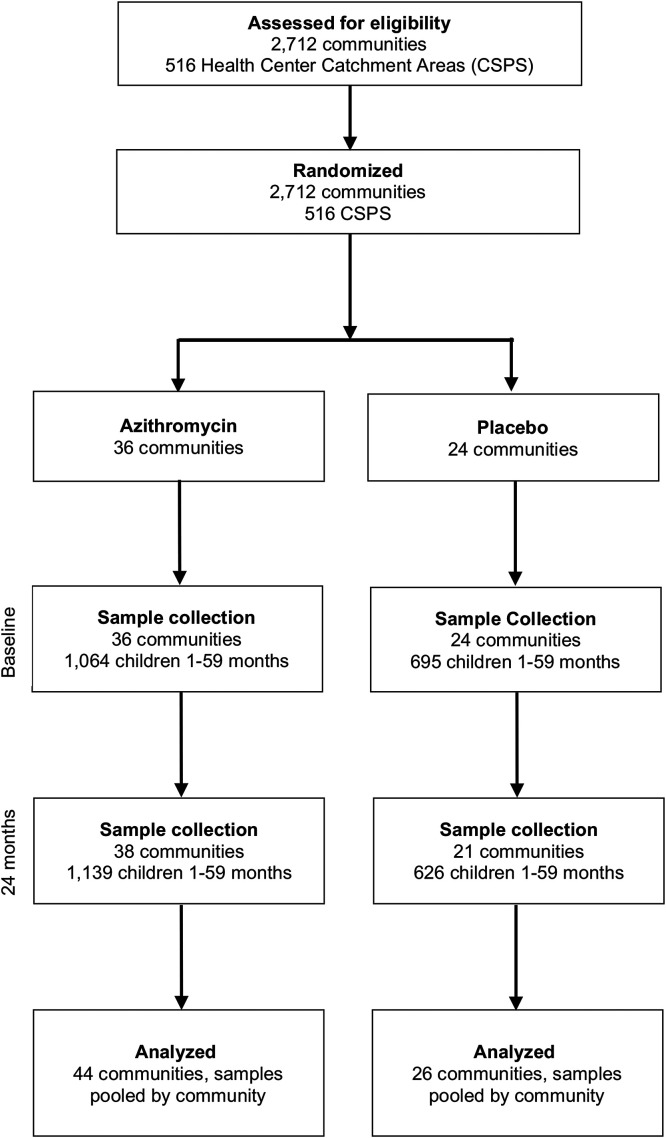
Consort diagram. CSPS = Centre de Santé et de Promotion Sociale.

**Figure 2.**
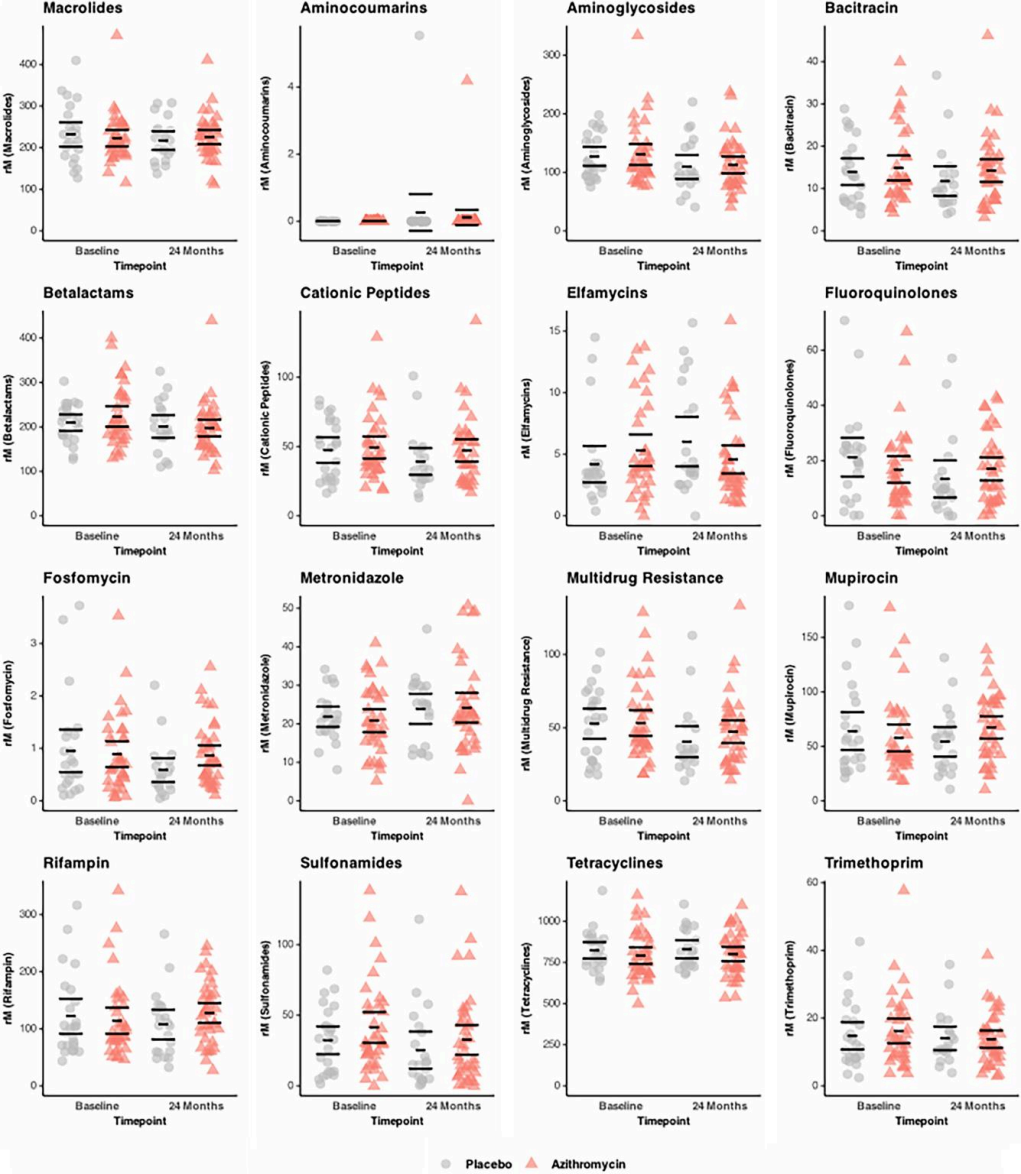
Normalized gut antibiotic resistance determinants of preschool children. Bars indicate the means and 95% CIs calculated by bootstrapping 1,000 times. Each point represents a community. Communities in which children ages 1–11 months old received placebo (*n* = 24 at baseline; *n* = 21 at 24 months) or azithromycin (*n* = 36 at baseline; *n* = 38 at 24 months) twice yearly are shown as circles and triangles, respectively. The *y* axes vary by antibiotic class to better display the data. rM = reads per million reads.

Among 516 participating communities, 60 communities were randomly selected for resistance monitoring at baseline (Figure 1). At 24 months, 59 communities were accessed, and the overall coverage across four rounds of treatment was 87.8% (SD = 13.9%) in the azithromycin group and 83.9% (SD = 12.1%) in the placebo group. At baseline, 36 communities randomized to azithromycin and 24 communities randomized to placebo were selected for AMR monitoring. The median age of the children in the azithromycin group was 24 months old (interquartile range [IQR]: 11–38 months old), and the median age of the children in the placebo group was 23 months old (IQR: 10–37 months old); 47% were female in the azithromycin group, and 50% were female in the placebo group (Table 1). At 24 months, 11 resistance monitoring communities had to be replaced because of logistical or security reasons. These replacement communities were chosen at random from the same pool. Ten of the 14 communities allocated for replacement were used, resulting in 38 communities treated with azithromycin and 21 communities treated with placebo sampled at 24 months. The remaining four communities allocated for replacement were not used to preserve the balance between treatment groups. At 24 months, children in the azithromycin group were a median of 19 months old (IQR: 11–31 months old), and the children in the placebo group were a median of 22 months old (IQR: 12–34 months old) (Table 1). In the azithromycin group, 47% were female, and in the placebo group, 50% were female. Rectal samples from 1,759 and 1,765 children were processed for DNA-seq at baseline and at 24 months, respectively (Figure 2).

**Table 1:** Demographics of children analyzed

Demographics	Rectal Swabs		
	Azithromycin	Placebo	Total
Baseline demographics			
No. of communities	36	24	60
No. of children	1,064	695	1,759
No. of children per communities, mean (SD)	29.6 (1.56)	28.96 (1.78)	29.32 (1.66)
Sex (%)			
Female	503 (47.3%)	348 (50.1%)	851 (48.4%)
Male	561 (52.7%)	347 (49.9%)	908 (51.6%)
Age, months			
Median (IQR)	24 (11–38)	23 (10–37)	23 (11–38)
24-Month demographics			
No. of communities	38	21	59
No. of children	1,139	626	1,765
No. of children per communities, mean (SD)	29.97 (1.24)	29.81 (1.21)	29.92 (1.22)
Sex (%)			
Female	531 (46.6%)	312 (49.8%)	843 (47.8%)
Male	608 (53.4%)	314 (50.2%)	922 (52.2%)
Age, months			
Median (IQR)	19 (11–31)	22 (12–34)	21 (11–33)

IQR = interquartile range.

**Figure 3.**
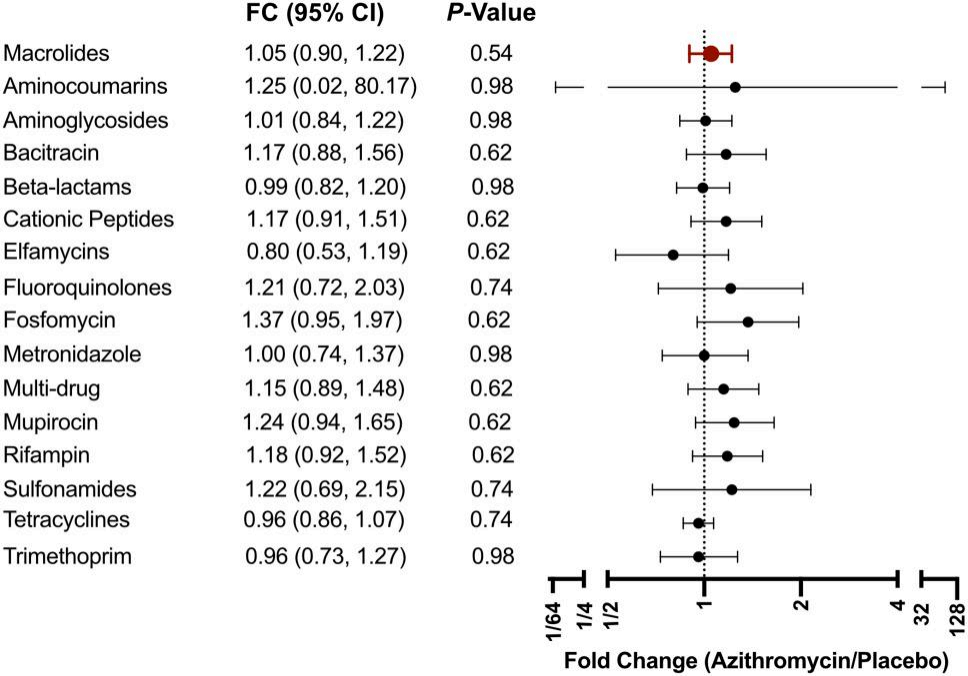
Change in antibiotic resistance determinants in the gut of preschool children between azithromycin- and placebo-treated communities at 24 months. Fold changes (FCs) in antibiotic resistance determinants in the azithromycin-treated group (*n* = 38) compared with the placebo-treated group (*n* = 21) with associated 95% CIs are shown. Macrolide resistance *P*-value was permuted with 10,000 simulations. *P*-values were obtained through the Wald test with Benjamini–Hochberg correction for nonmacrolide resistance multiple comparisons.

The gut macrolide resistance burden measured in children ages 1–59 months old in communities with infants treated with azithromycin was not statistically different from that in communities treated with placebo (1.05-fold, 95% CI: 0.90–1.22, *P* = 0.54) (Figures 2 and 3; Table 2). A sensitivity analysis was also performed, which adjusted for the community baseline load of macrolide resistance. The adjusted mean difference in the load of resistance determinants for macrolides between groups was 11.3 reads per million reads (95% CI: −17.8 to 40.4) and was not statistically significant (*P* = 0.22) (Supplemental Figure 1).

**Table 2:** Differential changes in macrolides resistance determinants between azithromycin and placebo groups at 24 months and differential changes in resistance determinants between azithromycin and placebo groups at 24 months

Class	Log_2_ Fold Change (95% CI)	Fold Change (95% CI)	*P*-Value
Macrolides	0.07 (–0.15 to 0.29)	1.05 (0.90–1.22)	0.54*
Aminocoumarins	0.32 (–5.68 to 6.33)	1.25 (0.02–80.17)	0.98^†^
Aminoglycosides	0.02 (–0.24 to 0.28)	1.01 (0.84–1.22)	0.98^†^
Bacitracin	0.23 (–0.18 to 0.64)	1.17 (0.88–1.56)	0.62^†^
Betalactams	−0.01 (–0.28 to 0.26)	0.99 (0.82–1.20)	0.98^†^
Cationic Peptides	0.23 (–0.13 to 0.59)	1.17 (0.91–1.51)	0.62^†^
Elfamycins	−0.32 (–0.90 to 0.26)	0.80 (0.53–1.19)	0.62^†^
Fluoroquinolones	0.27 (–0.48 to 1.02)	1.21 (0.72–2.03)	0.74^†^
Fosfomycin	0.45 (–0.08 to 0.98)	1.37 (0.95–1.97)	0.62^†^
Metronidazole	0.00 (–0.44 to 0.45)	1.00 (0.74–1.37)	0.98^†^
Multidrug resistance	0.20 (–0.17 to 0.56)	1.15 (0.89–1.48)	0.62^†^
Mupirocin	0.31 (–0.09 to 0.72)	1.24 (0.94–1.65)	0.62^†^
Rifampin	0.24 (–0.13 to 0.60)	1.18 (0.92–1.52)	0.62^†^
Sulfonamides	0.29 (–0.53 to 1.10)	1.22 (0.69–2.15)	0.74^†^
Tetracyclines	−0.06 (–0.22 to 0.09)	0.96 (0.86–1.07)	0.74^†^
Trimethoprim	−0.06 (–0.46 to 0.35)	0.96 (0.73–1.27)	0.98^†^

* The *P*-value was obtained through the Wald test and was not corrected for multiple comparisons.

^†^ Benjamin–Hochberg false discovery rate correction was performed for all antimicrobial classes.

Table 2 and Supplemental Figure 1 summarize the genetic resistant determinants for nonmacrolide antibiotic classes. Overall, there were no statistically significant differences between treatment groups for any of the antibiotic classes measured. In particular, the fold changes for aminoglycosides and betalactams were 1.01 (95% CI: 0.84–1.22, *P* = 0.98) and 0.99 (95% CI: 0.82–1.20, *P* = 0.98), respectively (Figure 3; Table 2). Adjustment for resistance determinants at baseline led to similar inference of no differences between groups in nonmacrolide classes (Supplemental Figure 1).

**Figure 4.**
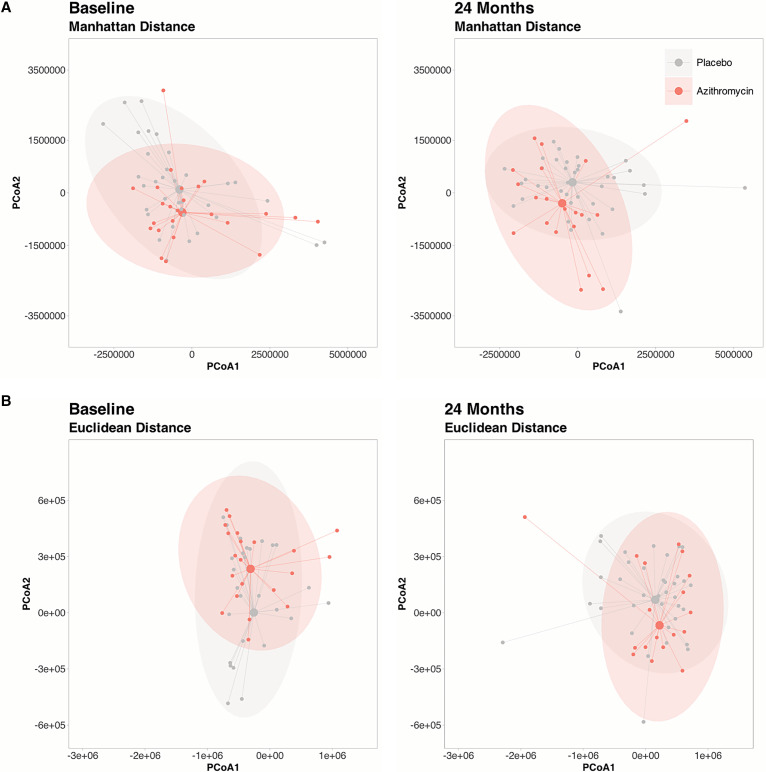
Gut microbiome community of preschool children in communities randomized to the placebo- and azithromycin-treated groups. Principal coordinates analyses (PCoAs) for Manhattan distance (**A**) and Euclidean distance (**B**) of the bacterial sequences aligned to the species level for placebo- and azithromycin-treated communities at baseline (*n* = 24 and *n* = 36, respectively) and 24 months (*n* = 21 and *n* = 38, respectively) are shown. Each point represents a community.

**Figure 5.**
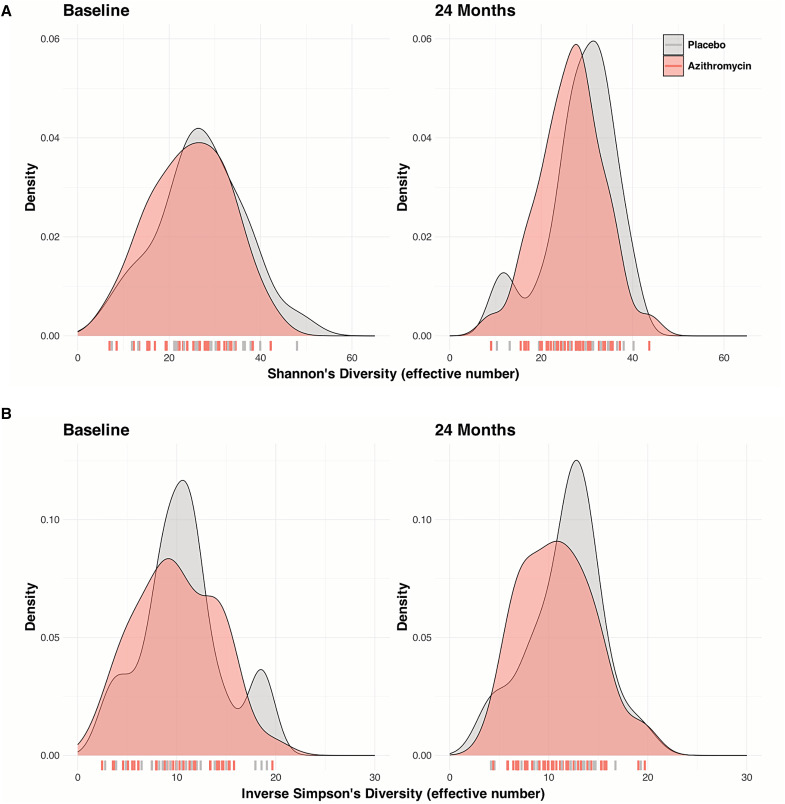
Gut microbiome diversity of preschool children in communities randomized to placebo- and azithromycin-treated groups. Density plots for Shannon diversity (**A**) and inverse Simpson diversity (**B**) at baseline (*n* = 24 for placebo; *n* = 36 for azithromycin) and at 24 months (*n* = 21 for placebo; *n* = 38 for azithromycin) are shown. The position of each community is shown in the bars below the density plots.

The gut microbiome structure of preschool children was similar at baseline (PERMANOVA Manhattan distance *P* = 0.29; PERMANOVA Euclidean distance *P* = 0.32) and remained similar at the 24-month time point (PERMANOVA Manhattan distance *P* = 0.46; PERMANOVA Euclidean distance *P* = 0.68) (Figure 4). Similarly, there was no difference in the gut microbiome diversity between azithromycin and placebo at baseline (Shannon alpha diversity: 24.64, 95% CI: 18.65–31.25 versus 26.32, 95% CI: 21.50–33.24, *P* = 0.44; inverse Simpson diversity: 9.79, 95% CI: 7.14–13.57 versus 10.58, 95% CI: 8.49–12.13, *P* = 0.61) (Table 3; Supplemental Table 1). Although the gut microbiome diversity appeared to decrease with azithromycin treatment at 24 months (Figure 5), the change was not statistically significant (Shannon alpha diversity: 27.12, 95% CI: 22.28–30.37 versus 29.74, 95% CI: 25.97–33.08, *P* = 0.16; inverse Simpson diversity: 10.62, 95% CI: 7.72–13.25 versus 12.23, 95% CI: 9.06–13.59, *P* = 0.31) (Table 3; Supplemental Figure 2; Supplemental Table 1).

**Table 3:** Descriptive statistics of the Shannon diversity index (effective number) by time point and treatment arm

Shannon Diversity Index	Azithromycin (*N* = 36)	Placebo (*N* = 24)	*P*-Value*
Baseline Shannon			
Median (IQR)	24.64 (18.65–31.25)	26.32 (21.50–33.24)	0.44
Mean (SD)	24.63 (8.64)	26.86 (9.52)	
End visit Shannon			
Median (IQR)	27.12 (22.28–30.37)	29.74 (25.97–33.08)	0.16
Mean (SD)	26.57 (6.94)	28.70 (7.58)	

IQR = interquartile range.

* *P*-values were obtained through the Wilcoxon rank sum test.

## DISCUSSION

In this double-masked, placebo-controlled, cluster-randomized trial, we compared the gut resistomes of preschool-aged children in communities that were randomized to have infants ages 1–11 months old treated with biannual azithromycin or placebo. We were unable to detect an increase in either macrolide or nonmacrolide resistance burden in the wider age range of children 1–59 months old. In addition, the gut microbiome structure and diversity of preschool children in the azithromycin communities were not distinguishable from those in the placebo-treated communities. These results are consistent with the minimal spillover effect of AMR in treated infants to older children in the community.^16^

Mass antibiotic distribution has been a key tool in trachoma elimination programs for the past 25 years.^17^^–^^19^ All individuals ages 6 months old and older are offered a single dose of azithromycin annually. These distributions clearly select for macrolide-resistant strains of bacteria in the nasopharynx and the gut.^18^^,^^20^ This resistance appears to decrease once the intervention is stopped, at least where tested.^21^ Childhood mortality programs have targeted treatments to preschool-aged children, with biannual distributions of oral azithromycin.^2^^,^^3^ Even these more limited distributions significantly increase macrolide resistance.^6^^,^^7^ Thus, WHO guidelines recommended restricting the intervention to infants ages 1–11 months old, still treating those most likely to benefit while limiting potential resistance.^8^ The recent Azithromycine pour la Vie des Enfants au Niger: Implementation et Recherche trial in Niger confirmed the efficacy of the treatment in children 1–59 months old while failing to prove the efficacy of just treating children 1–11 months old.^4^ In fact, the full effect of the intervention was only realized when the older children (12–59 months old) were also included, suggesting an indirect, herd-like effect on the community load of pathogens.^4^

Individual-randomized trials have found that a single dose of azithromycin selects for resistance in the first 2 weeks after treatment, although resistance was difficult to detect at 6 months.^9^ Mass treatment of children 1–11 months old may be more similar to individual treatment, where both the selection for macrolide resistance and alterations in the gut microbiome were transiently notable, but those changes returned to the untreated state by 6 months.^9^ Although it is possible that the transmission of gut pathogens carrying resistance determinants from these infants to their older siblings occurred in this study, such a spillover effect did not appear to be long lasting. It is worth noting that the number of eligible children being targeted for treatment in the current study (1–11 months old) was one fifth the number in prior studies, which targeted children 1–59 months old in the communities, and thus, the absence of an increase in macrolide and nonmacrolide resistance in the community was not surprising. Any increase in macrolide resistance may have been diluted with the inclusion of samples from children who were not targeted (12–59 months old). The results also suggested that there were minimal, if any, spillover effects from the infants to children 12–59 months old. Similarly, given that only one fifth of the children measured were treated in the current study, any differences in resistance in the children 1–11 months old may have gone undetected by 6 months after treatment.

A major strength of this study is its cluster-randomized study design, which leveraged the established CHD platform to reach eligible infants in the communities across large areas of Burkina Faso. The unbiased nature of DNA-seq allowed for the simultaneous interrogation of both the gut microbiome and multiple classes of AMR determinants in populations where the maintenance of cold chain can be challenging, even in the best circumstances. Conversely, a major limitation of this study is the lack of phenotypic evaluation of antibiotic-resistant pathogens in the gut. The clinical correlations between genetic mutations and phenotypic resistance in vivo are complex.^22^^–^^24^ Although it was reassuring that the gut resistomes were similar between communities treated with placebo compared with those treated with azithromycin, we cannot rule out mechanisms independent of genetic changes, such as biofilm formation.^22^ Other limitations include the lack of sample collection and assessment of the gut microbiome and resistome at shorter time points after treatment administrations and that the trial only included three regions of Burkina Faso. Generalization outside of the studied regions in Burkina Faso is likely to be limited.

## CONCLUSION

Overall, biannual azithromycin distributions to infants in Burkina Faso did not lead to detectable changes in AMR determinants in the gut or microbiome of older preschool-aged children in those same communities. These findings are consistent with the 2020 WHO guidelines, which assume the minimization of AMR in the communities when mass drug distribution is limited to the most vulnerable populations.^8^

## Supplemental Materials

10.4269/ajtmh.25-0238 Supplemental Materials
