# Supplementary material for: Effect of Biannual Azithromycin Distribution to Infants on Community Gut Resistome and Microbiome: A Cluster-Randomized, Controlled Trial
Source: Am J Trop Med Hyg. 2025 Oct 23;113(6):1311–9. doi: 10.4269/ajtmh.25-0238 (PMC12676621; doi:10.4269/ajtmh.25-0238)

Supplementary Table 1
Descriptive Statistics of Inverse Simpson's Diversity Index (Effective Number) by Timepoint and Treatment Arm

	**Azithromycin (N=38)**	**Placebo (N=21)**	***P*-value[a]**
**Baseline Inverse Simpson**			0.61
Median	9.79 (7.14, 13.57)	10.58 (8.49, 12.13)	
Mean (SD)	10.00 (4.12)	10.63 (4.23)	
**End Visit Inverse Simpson**			0.31
Median	10.62 (7.72, 13.25)	12.23 (9.06, 13.59)	
Mean (SD)	10.91 (3.71)	11.73 (3.68)	

[a]*P*-values were obtained through Wilcoxon Rank Sum Test. IQR = interquartile range, SD = standard deviation.

Supplementary Figure 1: Change in gut antibiotic resistance determinants at 24 months. Adjusted mean differences in antibiotic resistance determinants (rM) in the azithromycin-treated group (*N*=38) compared to the placebo-treated group (*N*=21) with associated 95% confidence interval (95% CI). All *P*-values were permuted with 10,000 simulations. Benjamini-Hochberg false discovery rate (FDR) correction was performed on permuted *P*-values for all other antibiotic classes except for macrolides. rM = reads per million reads.

Class	Mean Difference (95% CI)	P-Value
Macrolides	11.29 (-17.82, 40.40)	0.218
Aminocoumarins	-0.29 (-0.76, 0.18)	0.779
Aminoglycosides	2.38 (-24.61, 29.36)	0.652
Bacitracin	2.14 (-2.83, 7.12)	0.429
Betalactams	1.35 (-33.40, 36.10)	0.656
Cationic peptides	7.15 (-7.30, 21.61)	0.410
Elfamycins	-1.61 (-3.91, 0.69)	0.915
Fluoroquinolones	4.63 (-3.55, 12.81)	0.410
Fosfomycin	0.31 (-0.04, 0.65)	0.410
Metronidazole	2.74 (-3.02, 8.50)	0.410
Multidrug resistance	5.54 (-8.93, 20.01)	0.429
Mupirocin	11.18 (-7.43, 29.79)	0.410
Rifampin	16.97 (-16.73, 50.67)	0.410
Sulfonamides	5.70 (-14.01, 25.41)	0.483
Tetracyclines	-20.96 (-96.63, 54.72)	0.779
Trimethoprim	-0.35 (-5.14, 4.43)	0.714

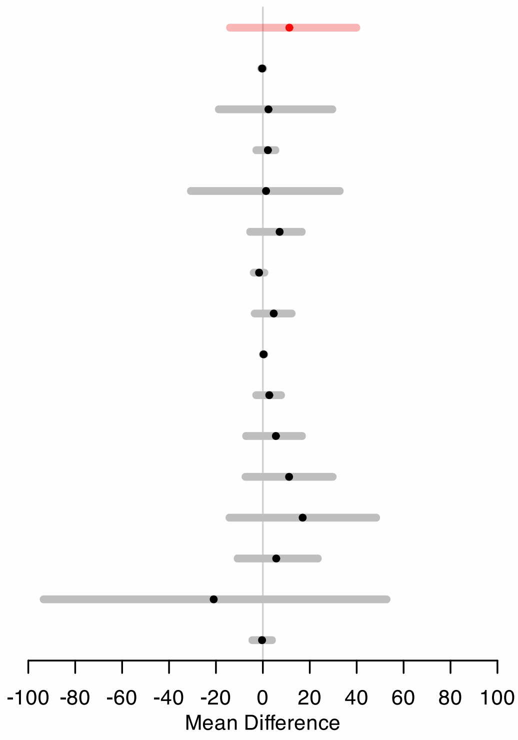

Supplementary Figure 2: Gut microbiome diversity of children in communities randomized to placebo and azithromycin. Scatter plot for Shannon's diversity index (A) and inverse Simpson's index (B) at baseline (*N*=24 for placebo; *N*=36 for azithromycin) and at 24 months (*N*=21 for placebo; *N*=38 for azithromycin). Each point represents a community. Error bars are median and 95% confidence intervals.

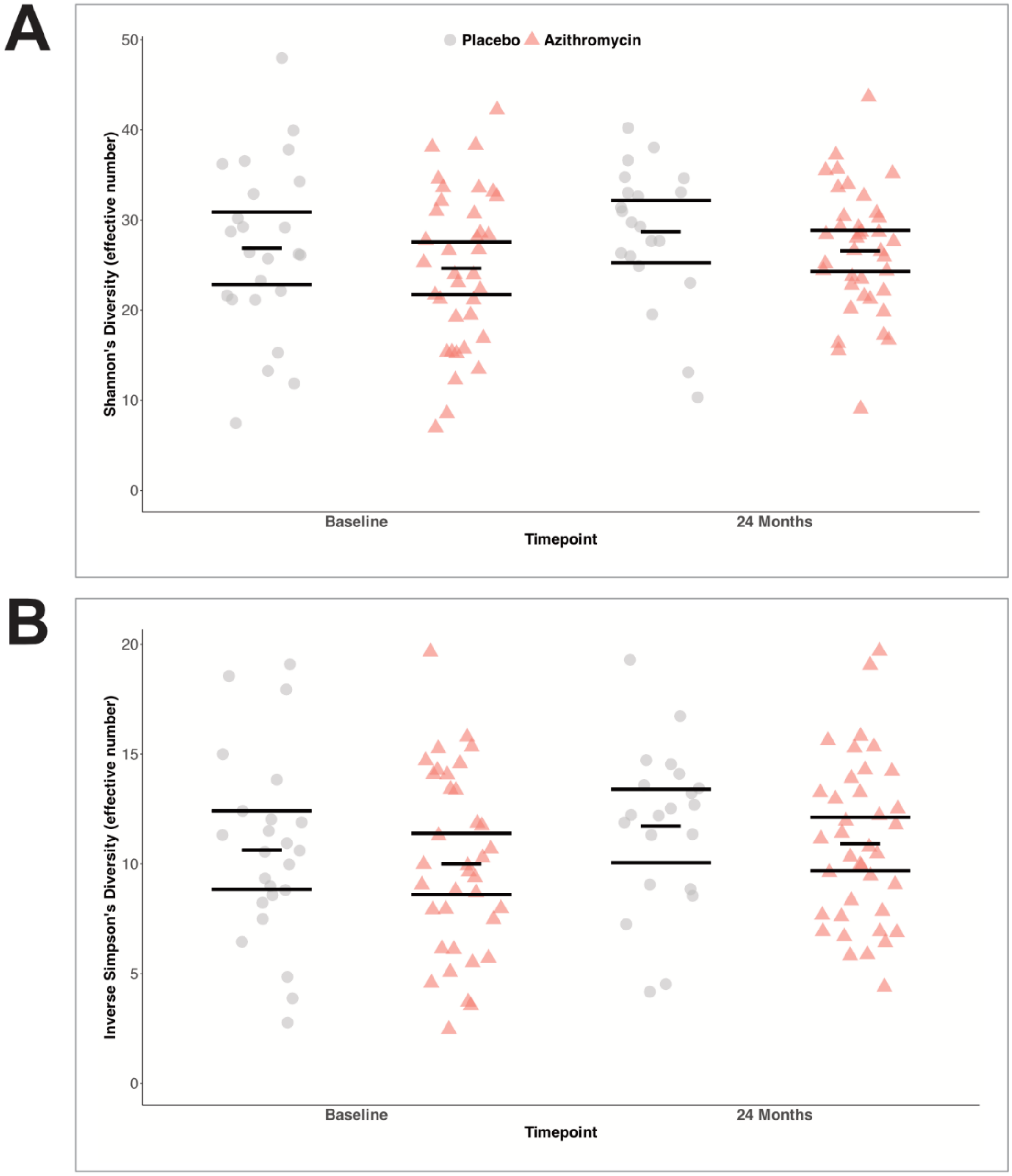

Supplement: Supplemental Materials [file tpmd250238.SD1.pdf]
